# Health Risk Assessment of Residents’ Exposure to Air Pollutants around the Sinpyeong–Jangrim Industrial Complex in Busan

**DOI:** 10.3390/toxics12090682

**Published:** 2024-09-20

**Authors:** Ji-Yun Jung, Jong-Won Kim, Tae-Woo Koo, Joon-Young Heo, Ye-Seul Jeong, Cheol-Min Lee

**Affiliations:** 1 Department of Chemical and Environmental Engineering, Seokyeong University, Seoul 02713, Republic of Korea; jju1049@skuniv.ac.kr; 2 Intelligent Machinery & Parts Center, Busan Technopark, Busan 46742, Republic of Korea; jwkim@btp.or.kr (J.-W.K.); twkoo@btp.or.kr (T.-W.K.); 3 Chemical Analysis Center, Korea Conformity Laboratories, Seoul 08503, Republic of Korea; heoyoung@kcl.re.kr (J.-Y.H.); seuljy@kcl.re.kr (Y.-S.J.)

**Keywords:** fine particulate, volatile organic compounds, heavy metals, industrial complex, health effects, risk assessment

## Abstract

In this study, we aimed to evaluate the health effects of heavy metals and volatile organic compounds (VOCs) in fine particulate matter (PM_2.5_) in the air around the Sinpyeong–Jangrim Industrial Complex, Busan, and the necessity of reduction measures. We measured the concentrations of heavy metals and VOCs in PM_2.5_ in five areas with dense population around the Sinpyeong–Jangrim Industrial Complex. A health risk assessment was conducted, and the spatial risk distribution for the entire Saha-gu area was predicted using inverse distance weighting (IDW). Our results indicated that the carcinogens benzene, As, and Cr^6+^, and the non-carcinogen m,p-xylene, have the potential to adversely affect health. In particular, As was found to have the potential to express health effects at all measurement points. Additionally, based on the IDW results, the minimum values of benzene, arsenic, and m,p-xylene exceeded the threshold level, potentially affecting the health of residents across the entire Saha-gu area. In contrast, Cr^6+^ could potentially impact health only in some parts of Saha-gu as only its maximum value exceeded the threshold level. We demonstrated the importance of reducing air pollutant emissions from general industrial complexes. Our results will be useful in establishing more systematic mitigation measures to protect the health of residents near the Sinpyeong–Jangrim Industrial Complex and developing air pollutant management systems.

## 1. Introduction

According to the National Institute of Chemical Safety (NICS), as of 2022, the amount of chemicals emitted from industrial complexes accounts for approximately 63.5% of total domestic emissions (65,212 tons) [1]. This indicates a high likelihood of harmful air pollutants entering residential areas near industrial complexes. Among these harmful air pollutants, volatile organic compounds (VOCs) and heavy metals are of particular concern owing to their high toxicity and carcinogenicity, posing significant health risks with long-term exposure, thus necessitating strict air quality management [2]. VOCs are those organic compounds with a Reid vapor pressure of over 10.3 Pa at normal temperature (293.15 K) and pressure (101.325 kPa) [3]. VOCs are a large group of carbon-based chemicals that easily evaporate at room temperature, and when exposed to the human body, they are absorbed directly into the lungs and exert toxic effects through the bloodstream [3,4]. Heavy metals, characterized by a density exceeding 5 g/cm^3^, can interact with specific compounds in the body, such as oxygen, to elicit toxic effects [5,6]. Some of the heavy metals are essential nutrients for life to maintain normal physiological functions, but they are toxic at high levels. When harmful heavy metals enter the body, they cause digestive system toxicity, kidney toxicity, neurotoxicity, etc., and arsenic and cadmium can also act as endocrine disruptors [7].

As industrialization and urbanization progress simultaneously, the population influx around industrial complexes has increased, leading to the development of residential areas adjacent to and intermingled with industrial zones, thus highlighting the need for health impact assessments in such areas [8,9]. Although numerous health impact studies have been conducted on residents near national industrial complexes [10,11], health impact assessments for residents near general industrial complexes, which constitute 55% of the industrial complexes in the country, are insufficient [2].

Busan has the highest number of industrial complexes among metropolitan cities in Korea, with 39 complexes, including one national industrial complex, 32 general industrial complexes, 5 urban high-tech industrial complexes, and 1 agro-industrial complex [12]. Among them, the Sinpyeong–Jangrim Industrial Complex, established in 1990, has been in operation for over 30 years and houses approximately 600 businesses, with over 30% of these businesses involved in dyeing, leather processing, and plating [13,14]. The Sinpyeong–Jangrim Industrial Complex is one of the most frequently cited sources of pollution complaints in Busan, and according to the Ministry of Environment, it is a high-priority area for environmental health assessment owing to reported health issues among nearby residents [15,16]. Additionally, a report from the Busan Development Institute [17] stated that Saha-gu, where the Sinpyeong–Jangrim Industrial Complex is located, is a vulnerable residential area owing to the emission of air pollutants, emphasizing the need for mitigation and management strategies based on a health impact assessment of the Sinpyeong–Jangrim Industrial Complex and its surrounding areas.

Against this backdrop, in this study, we aimed to measure the concentrations of heavy metals and VOCs in fine particulate matter (PM_2.5_) in the air near the Sinpyeong–Jangrim Industrial Complex in Saha-gu, Busan to determine their spatial concentration distribution characteristics, and assess the health risks for residents near the industrial complex. We aimed to confirm the necessity of an environmental health impact assessment for the Sinpyeong–Jangrim Industrial Complex and its surrounding residential areas, and accordingly develop mitigation and management strategies.

## 2. Materials and Methods

### 2.1. Measurement Sites

**Figure 1:**
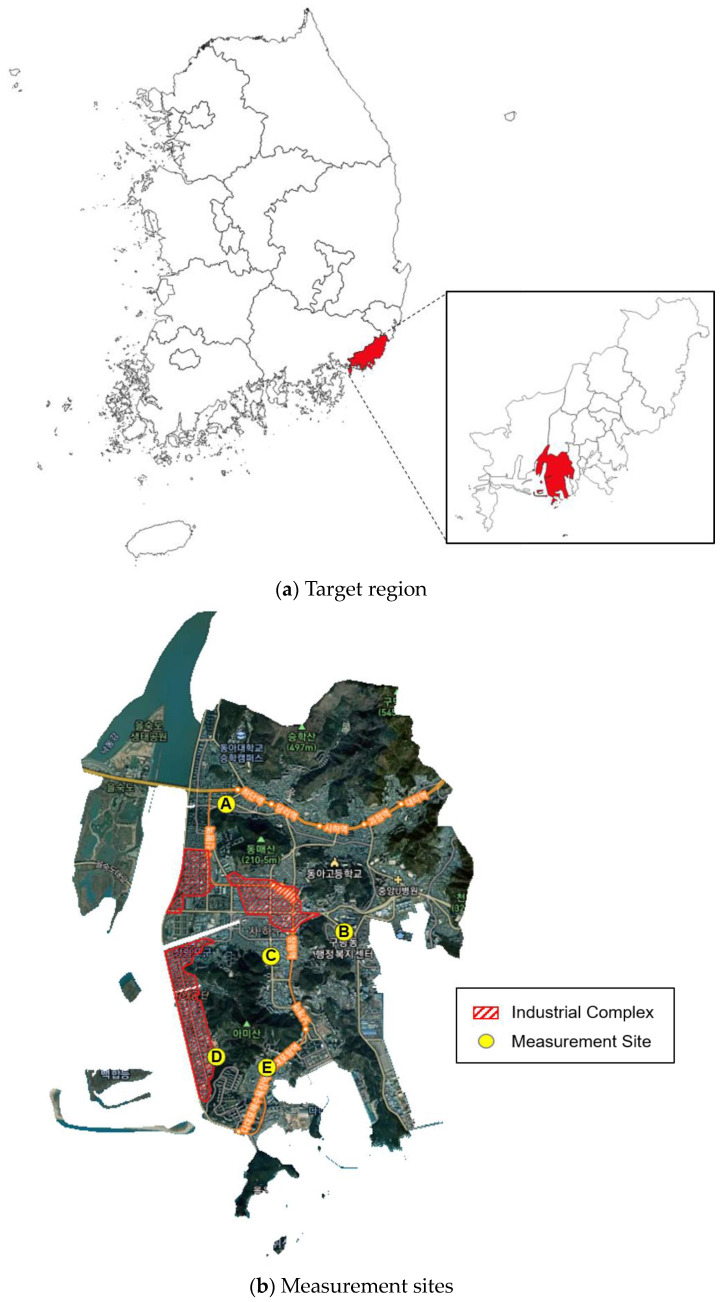
Target area and measurement sites (top is (**a**) and bottom is (**b**)).

In this study, Saha-gu, a place with a high frequency of cases exceeding the fine dust concentration standard, was selected in Busan, and Sinpyeong–Jangrim Industrial Complex located in Saha-gu was selected as the subject of the study. To assess and compare the health impacts of exposure to heavy metals and VOCs in PM_2.5_ on residents living near the Sinpyeong-Jangrim Industrial Complex in Saha-gu, Busan, we selected five residential areas as measurement sites, with dense populations near the industrial complex, as shown in Figure 1. The measurement sites were chosen by considering the predominant wind direction and geographical characteristics of the Sinpyeong–Jangrim Industrial Complex, application of the inverse distance weighting (IDW) method, elevation of buildings, local cooperation, and availability of electricity. These factors ensured that the selected sites were appropriate for evaluating the influence of harmful pollutants emitted from the industrial complex and assessing the air quality in the residential areas surrounding the measurement sites.

### 2.2. Sampling and Analysis

Sample collection was conducted over approximately one year from December 2022 to December 2023. For heavy metals, sampling was conducted twice a week during the winter and spring seasons, with high particulate matter concentrations and significant yellow dust influences, and once a week during the summer and fall seasons. VOCs were measured at the same frequency as heavy metals: twice a week in winter and spring, and once a week in summer and fall [18,19]. The heavy metals in PM_2.5_ were measured using a PM_2.5_ sampler (LAS-16, APM Co., Bucheon-si, Republic of Korea), designed according to the United States Environmental Protection Agency Federal Reference Methods (US EPA FRM) for PM_2.5_ measurement. The sampler was operated at a flow rate of 16.7 L/min, collecting PM_2.5_ over a 24 h period from 00:00 to 23:59 the following day and using Teflon filters (PTFE 2.0 μm, ∅47 mm) for heavy metal analysis. VOCs were measured using an air sampler (MP-∑30KNII, SIBATA Co., Soka Saitama, Japan) at a flow rate of 100 mL/min, with each sample being collected over 30 min. Samples were collected twice daily: once between 9:00 and 12:00 and once between 12:00 and 15:00, with a total of two samples per day [20].

Sample collection and transportation were conducted according to the guidelines of the National Institute of Environmental Research for the installation and operation of air quality monitoring networks [20]. For heavy metals in PM_2.5_, the filters were placed in Teflon containers, followed by the addition of a mixed acid solution of 5.5% nitric acid and 16.7% hydrochloric acid. The Teflon containers with the acid solution were maintained at 180 °C for 10 min using a 1200 W microwave. After filtration, the final volume was diluted to 25 mL in a volumetric flask. The concentrations of each heavy metal were than analyzed using inductively coupled plasma (ICP) spectrometry by measuring the emission intensity at their characteristic wavelengths. For VOCs, the collected solid sorbent tubes were sealed in aluminum foil, placed in an icebox below 4 °C, and transported. The solid sorbent tubes were then thermally desorbed in a thermal desorption unit, concentrated in a low-temperature trap, and subsequently thermally desorbed. The samples were then analyzed using gas chromatography/mass spectrometry (GC/MS).

### 2.3. Quality Assurance/Quality Control

To verify the reliability of the analysis results, the quality control of the measurement analysis (QA/QC) was conducted, and the Method Detection Limit (MDL) and Relative Standard Deviation (RSD) were calculated according to the guidelines of the National Institute of Environmental Research for the installation and operation of air quality monitoring networks. Table 1 shows the results of the quality control.

### 2.4. Health Risk Assessment

The toxicity information for heavy metals and VOCs in PM_2.5_ was surveyed using the US EPA’s Integrated Risk Information System (IRIS). The unit risk (UR) and reference concentration (RfC) for each substance were examined. The health risk assessment of exposure to heavy metals and VOCs in PM_2.5_ was focused on five heavy metals (Ni, As, Cd, Cr^6+^, and Mn) and six VOCs (benzene, toluene, ethylbenzene, m,p-xylene, o-xylene, and styrene); their toxicity information can be obtained from the US EPA. The toxicity data for these heavy metals and VOCs are summarized in Table 2 and Table 3.

The toxicity values for carcinogens were converted from UR to the carcinogenic potency factor (CPF) by considering the body weight and respiratory rate (Equation (1)). The CPF represents the risk for an adult exposed to the pollutant at a breathing rate of 20 m^3^/day over a 70-year lifetime.

(1) $$CPF[{\left(mg/kg/day\right)}^{-1}]=\frac{Unit\;Risk{\left(\mu g/m^{3}\right)}^{-1}\times 70\;kg}{20\;m^{3}/day}\times 1000\;\mu g/mg$$

For non-carcinogens, the toxicity values were converted from RfC to a reference dose (RfD) by considering the body weight and respiratory rate (Equation (2)).

(2) $$RfD(mg/kg/day)=\frac{RfC(mg/m^{3})\times 20(m^{3}/day)}{70(kg)}$$

As the sampling points were located around Busan’s Sinpyeong–Jangrim Industrial Complex, the health risk assessment targeted residents living nearby, considering only inhalation exposure. The assessment was conducted for adults aged 19 and above. The life expectancy was set to 82.7 years based on data from 2023 from the Korea Statistical Information Service [36], and the average exposure period was calculated from 19 to 82.7 years (in days). An annual average of 300 exposure days (standard deviation of 19 days) and a maximum of 330 days were assumed [37]. The exposure factors used in this assessment are summarized in Table 4.

For the heavy metal Cr^6+^, 8% of the measured total Cr concentration was considered in the exposure assessment. This percentage reflects a conservative estimate, combining the average Cr^6+^ ratio in the total Cr concentration observed in domestic industrial complexes (0.7–2.4%) and urban areas abroad (3–8%) [39].

The lifetime average daily dose (LADD) for carcinogens was calculated using the air concentration of each substance and exposure factor, whereas the average daily dose (ADD) was calculated for non-carcinogens (Equations (3) and (4)). LADD and ADD assume that 100% of the exposed amount is absorbed by the human body.

(3) $$LADD(mg/kg/day)=\frac{C\times IR\times ED}{BW\times LT}$$

(4) $$ADD(mg/kg/day)=\frac{C\times IR\times ED}{BW\times AT}$$

where C is the substance concentration (mg/m^3^), IR is the inhalation rate (m^3^/day), ED is the exposure duration (days), BW is the body weight (kg), LT is the lifespan (days), and AT is the average exposure time (days).

The excess cancer risk (ECR) for carcinogens was determined by multiplying the LADD with CPF (Equation (5)). We set an ECR threshold of 1 × 10^−5^, and any value above this threshold indicates a potential health risk from exposure. Although the principle threshold for ECR is 1 × 10^−6^, a threshold of 1 × 10^−5^ is used for establishing mitigation measures in health and environmental impact assessments [40]. For non-carcinogens, the hazard quotient (HQ) was calculated by dividing the ADD by RfD (Equation (6)). An HQ exceeding 1 suggests a potential health risk from exposure [41].

(5) ECR=LADD×CPF

(6) $$HQ=\frac{ADD}{RfD}$$

### 2.5. Risk Prediction Using IDW

IDW is a spatial interpolation method based on geographic space. It is used to calculate the attribute values of unknown points by assuming that while the attributes of any two points are related, their similarity is inversely correlated with the distance between them [42,43]. In this study, IDW was used to predict the spatial concentration distribution of target substances across the entire Saha-gu district of Busan, based on the concentrations measured at five sampling points. The spatial distribution was modeled in 50 × 50 m grids. Additionally, the predicted concentrations were used to estimate the spatial distribution of risk for each target substances. The health risk for each substance was calculated using the health risk assessment method employed in this study. The resulting spatial risk distribution was visualized as a risk map using the open source Geographic Information System (GIS) program Quantum Geographic Information System (QGIS ver 3.36.2) [44].

## 3. Results

### 3.1. Health Risk Assessment

Table 5 presents the mean value and standard deviations for the five selected heavy metals and six VOCs. For a conservative assessment, the reasonable maximum exposure (RME) was determined by adding three times the standard deviation to the mean value, and this RME was used for a conservative health risk assessment. Also, an analysis of variance (ANOVA) was performed to compare the concentration among the measurement sites. Statistical significance level was set at *p* < 0.05. It was found that Cd, Cr, and Toluene concentrations significantly differed among the measurement sties (*p* < 0.05). The post hoc test results revealed that the Cd concentration in sites C, D, and E differed from that in other sites. Moreover, Cr concentrations in sites B and D differed from that in other sites, and Toluene concentrations in sites A, B, and C differed from that in other sites.

**Figure 2:**
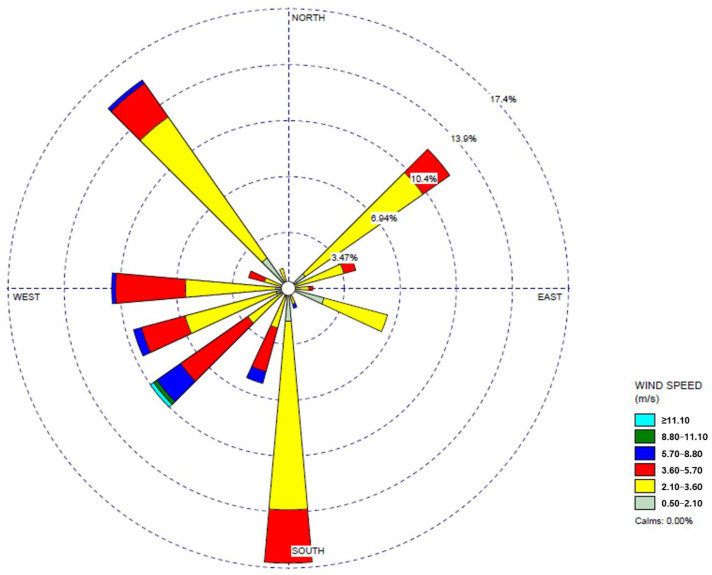
Wind rose results for the measurement period in Saha-gu, Busan.

The health risk assessment for carcinogenic substances, including benzene, Ni, As, Cd, and Cr^6+^, was conducted by calculating the ECR, as presented in Table 6. Benzene and As exceeded the cancer risk threshold at both the central tendency exposure (CTE) and RME levels across all measurement sites, indicating a potential health impact from these substances. Therefore, benzene and As should be prioritized in air quality management areas. For Cr^6+^, although only point D exceeded the cancer risk threshold at the CTE level, all five measurement sites exceeded the threshold at the RME level, indicating a potential health risk. Point D, being the closest residential area to the Sinpyeong–Jangrim Industrial Complex, suggests that reducing Cr^6+^ emissions within the industrial area could positively impact the surrounding air quality. Thus, controlling potential Cr^6+^ sources within the industrial complex should be prioritized. For Ni, no measurement sites exceeded the health impact threshold at the CTE level. However, at the RME level, points D and E exceeded the cancer risk threshold. Despite Ni having relatively lower priority compared with other carcinogenic pollutants, the areas with Ni exceeding the threshold (points D and E) are close to the Sinpyeong–Jangrim Industrial Complex and are downwind of the prevailing wind direction, as shown in Figure 2. This underscores the need for ongoing monitoring and evaluation of Ni emissions from the industrial complex and their impact on nearby areas.

The health risk assessment for non-carcinogenic substances, including benzene, toluene, ethylbenzene, m,p-xylene, o-xylene, styrene, Mn, and Cr^6+^, was conducted by calculating the HQ as presented in Table 7. At the CTE level, only m,p-xylene exceeded the non-cancer risk threshold at points B and C. At the RME level, m,p-xylene in all five measurement sites exceeded the threshold, indicating that it must be prioritized for management among the non-carcinogenic pollutants. Considering that points B and C are located in the central-eastern part of the Sinpyeong–Jangrim Industrial Complex, the management plans must focus on the sources in this area. Notably, o-xylene, Mn, and benzene did not exceed the non-cancer risk threshold at the CTE level. However, at the RME level, o-xylene and Mn exceeded the threshold at all points except A, and benzene exceeded the threshold only at point B, indicating the need for continuous monitoring and evaluation.

### 3.2. Spatial Risk Distribution and Creation of a Risk Map Using IDW

**Figure 3:**
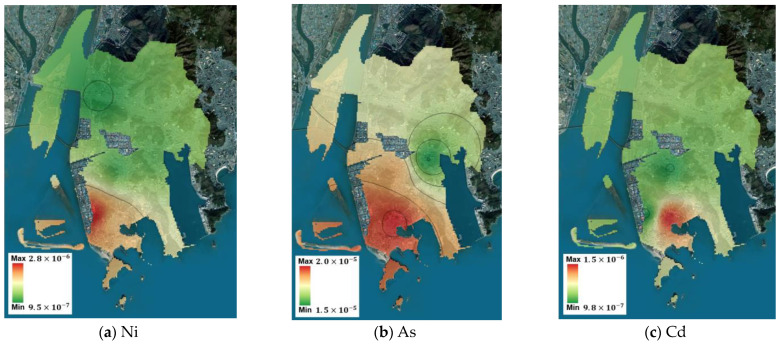

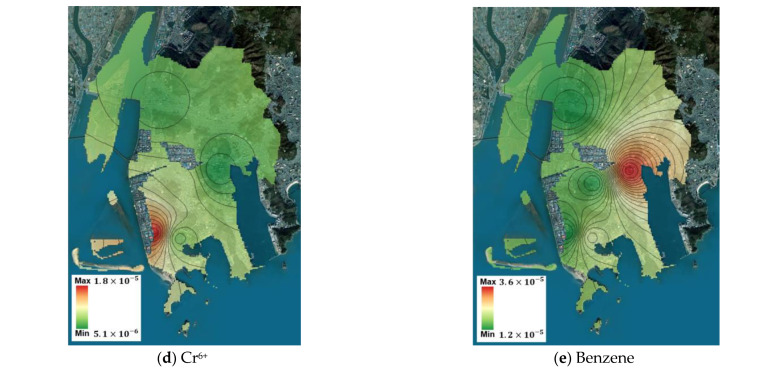
Maps of carcinogenic risk (CTE) for each target substance (**a**–**e**).

**Figure 4:**
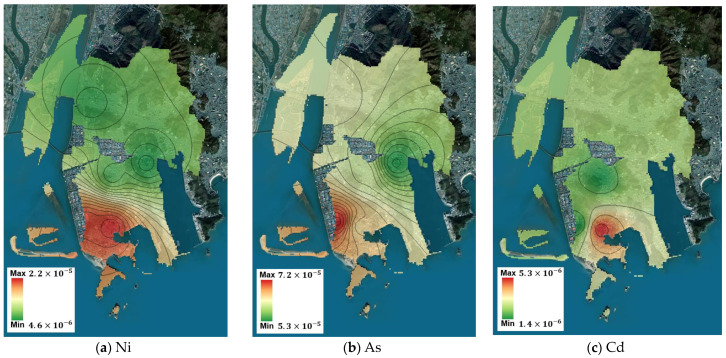

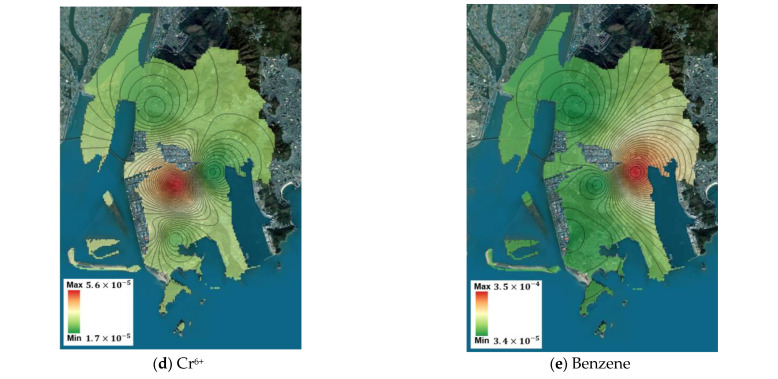
Maps of carcinogenic risk (RME) for each target substance (**a**–**e**).

**Figure 5:**
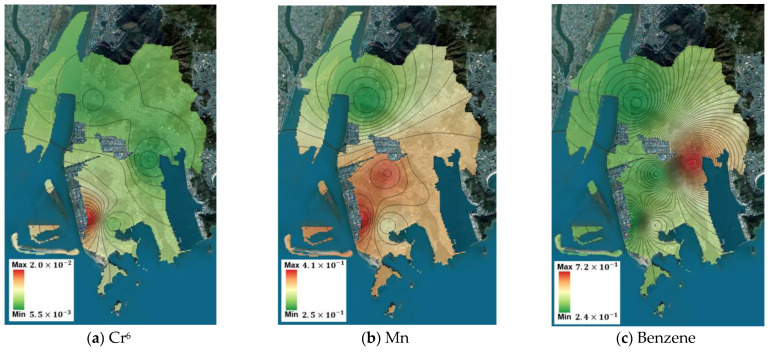

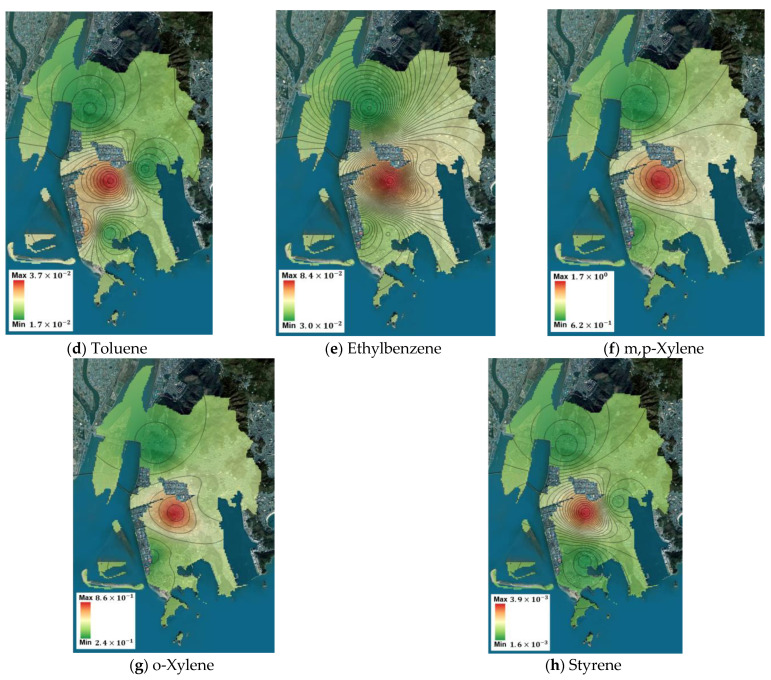
Maps of non-carcinogenic risk (CTE) for each target substance (**a**–**h**).

**Figure 6:**
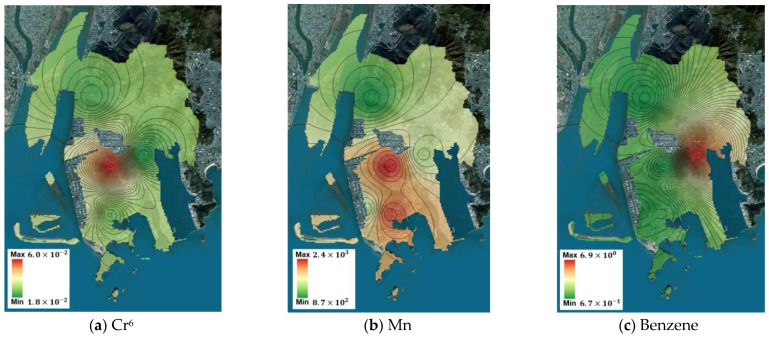

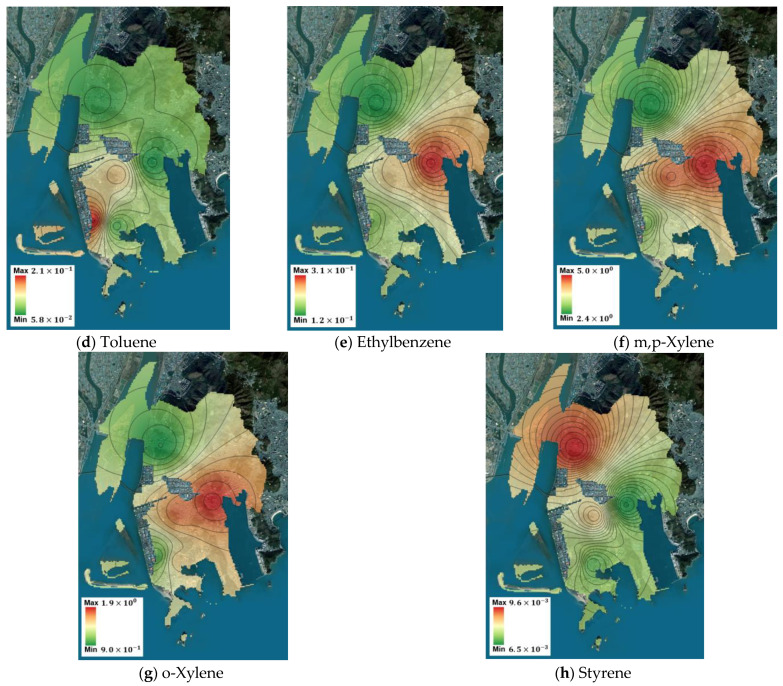
Maps of non-carcinogenic risk (RME) for each target substance (**a**–**h**).

Using the measurement data from the five points, the spatial concentration distribution of the target substances throughout Saha-gu was predicted using IDW. Based on this prediction, the spatial risk distribution for Saha-gu was calculated, and the maximum and minimum values are summarized in Table 8. For carcinogenic substances, benzene and As had minimum values exceeding the ECR threshold of 1 × 10^−5^, indicating high potential for health impacts across Saha-gu, thereby reaffirming the need for prioritized management plans for these substances. Although Cr^6+^ did not exceed the threshold at its minimum value, its maximum value exceeded the threshold, suggesting localized potential health impacts. For non-carcinogenic substances, the minimum and maximum values of m,p-xylene were lesser and greater than the threshold, respectively, indicating localized potential health impacts. The spatial concentration predictions and corresponding spatial risk distributions for the Sinpyeong–Jangrim Industrial Complex and its surrounding areas in Saha-gu were used to create risk maps (Figure 3, Figure 4, Figure 5 and Figure 6). These maps demonstrate that when developing air quality management plans for Saha-gu, customized air quality management strategies tailored to specific pollutants and areas must be implemented rather than implementing a uniform approach for the entire region.

## 4. Discussion

In this study, we evaluated the need to establish management measures through health risk assessments of air pollutants near the Sinpyeong–Jangrim Industrial Complex in Busan. By investigating the concentrations of heavy metals and VOCs within PM_2.5_ at five locations around residential areas near the industrial complex, health risk assessments for each measurement site were conducted. Furthermore, using the IDW interpolation method, the spatial distribution of harmful pollutants and corresponding risk distribution maps were established to confirm the impact on the health of residents and need for management measures.

The health risk assessment for heavy metals in PM_2.5_ indicated that As posed potential health risks at all measurement sites. The average concentration of As measured in this study (8.47 ng/m^3^) was approximately 2.2 times higher than that (3.85 ng/m^3^) in Hakjang-dong, located in the Sasang Industrial Complex of Busan [45]. This discrepancy between As concentrations in this study and Hakjang-dong is likely attributable to differences in the number and types of industries within the Sinpyeong–Jangrim and Sasang Industrial Complexes. Although As is primarily emitted from metal smelting industries, it can also be released from processes involving air pollution control equipment and heat treatment. Thus, even after the installation of control equipment and heat treatment, As requires continuous management [46]. Considering these findings, air quality management plans must be established for the continuous monitoring and reduction in As around the Sinpyeong–Jangrim Industrial Complex in Saha-gu, Busan. Cr^6+^ posed potential health risks only at point D, where its concentration was found to be more than twice as high as that at other points. The Cr^6+^ concentration at point D was at least 1.5 times higher than that measured in adjacent areas, such as Yeonsan-dong (6.8 ng/m^3^), Hakjang-dong (1.6 ng/m^3^), and Deokcheon-dong (2.0 ng/m^3^) [47,48,49]. These results suggest that point D, being the closest residential area to the Sinpyeong–Jangrim Industrial Complex, should be prioritized for Cr^6+^ source management over the broader Saha-gu area.

The health risk assessment for VOCs revealed that benzene, a well-known carcinogen, posed potential health risks at all measurement sites. Benzene is used across various industries in the Sinpyeong–Jangrim Industrial Complex, including petroleum refining, primary metal manufacturing, and electronics and automobile manufacturing [50]. Yoon et al. (2021) [2] reported higher benzene exposure levels in residents near the Sinpyeong–Jangrim Industrial Complex compared with those in Deokcheon-dong; this underscores the necessity for health impact assessments and subsequent mitigation and management plans for benzene emissions from the industrial complex. The health risk assessment indicated that m,p-xylene posed potential health risks at points B and C. This result is likely ascribable to the presence of numerous industries, such as transportation equipment manufacturing, electronic components and computer manufacturing, and rubber and plastic product manufacturing, which use xylene as a paint thinner or solvent [51] in the areas surrounding points B and C. However, Cheong and You (2011) [52] stated that the main sources of VOCs in Saha-gu are gas stations and garages. Thus, the measurement sites, being close to residential areas, may also be influenced by non-industrial sources.

Examining the predicted risks and risk maps generated using IDW, we found that the minimum values of both As and benzene (carcinogens) exceeded the threshold, suggesting a potential health impact across Saha-gu. Both substances were found to pose health risks at all measurement sites based on health risk assessments. Considering that the International Agency for Research on Cancer (IARC) classifies these substances as Group 1 carcinogens and that they are toxic to various organs, including the cardiovascular and nervous systems, focused management is essential [53,54]. The maximum values of Cr^6+^ and m,p-xylene exceeded the threshold values under CTE, indicating potential localized health impacts. Under RME, even their minimum values exceeded the threshold, suggesting potential health impacts across Saha-gu. Notably, xylene ranks as the sixth most frequently handled substance in Saha-gu industries based on statistics from the National Institute of Chemical Safety. This underscores the need for comprehensive management plans encompassing the entirety of Saha-gu, including the Sinpyeong–Jangrim Industrial Complex [55].

In this study, we conducted a health risk assessment of exposure to heavy metals and VOCs in PM_2.5_ near the Sinpyeong–Jangrim Industrial Complex and highlighted the need for measures to reduce air pollutant emissions. However, our study has some limitations. First, owing to the lack of specific data on body weight and respiration rates for residents near the industrial complex, the general exposure coefficients for Koreans were applied, which could result in underestimation or overestimation. Second, we only conducted deterministic risk assessments without considering uncertainties in each step. Third, the limited data on heavy metals and VOCs in the measurement period increased the uncertainty in concentration estimates. Despite these limitations, we demonstrated the importance of reducing air pollutant emissions in general industrial complexes, which are often neglected compared with national industrial complexes. Moreover, by using IDW, we not only assessed health impacts at specific measurement sites but also predicted and evaluated health impacts across Saha-gu, providing a basis for developing tailored management plans for different areas.

## 5. Conclusions

In this study, we aimed to assess the health impacts of heavy metals and VOCs in PM_2.5_ in the vicinity of the Sinpyeong–Jangrim Industrial Complex in Busan, and identify the need for mitigation measures. From December 2022 to December 2023, we investigated the concentrations of heavy metals and VOCSs in PM_2.5_ around the Sinpyeong–Jangrim Industrial Complex, conducted a health risk assessment for nearby residents, and used IDW to predict the overall risk in Saha-gu, Busan.

The health risk assessment results indicated that at average levels, benzene, As, and Cr^6+^ exceeded the threshold of 1 × 10^−5^, indicating potential health risks. At the RME level, benzene, As, Cr^6+^, and Ni were found to pose potential health risks. Among non-carcinogenic substances, only m,p-xylene exceeded the threshold of 1 at average levels, indicating potential health risks. At the RME level, benzene, m,p-xylene, o-xylene, and Mn posed potential health risks.

The IDW results predicted that the minimum values of As and benzene exceeded the threshold for carcinogens, indicating potential health risks across Saha-gu. For Cr^6+^, only the maximum values exceeded the threshold, suggesting potential health risks in specific areas of Saha-gu. For non-carcinogenic substances, the maximum values of m,p-xylene exceeded the threshold, indicating potential health risks in specific areas of Saha-gu.

The significance of this study is that we conducted a health risk assessment of heavy metals and VOCs in PM_2.5_ around the Sinpyeong–Jangrim Industrial Complex and predicted the overall risk for Saha-gu, including areas that were not directly measured. Our results highlight the need for systematic mitigation measures to protect the health of residents near the Sinpyeong–Jangrim Industrial Complex. Also, it is considered that one control method is to investigate information on emissions by business sites and install reduction facilities that can reduce substances. The findings are expected to be valuable for developing an air pollution management system for the entirety of Saha-gu.

## Figures and Tables

**Table 1:** The results of the quality assurance and quality control.

Pollutants		MDL ^a^	RSD ^b^ (%)
Heavy metals (ng/m^3^)	Ni	4.07	0.28
	As	1.37	0.31
	Cd	7.69	1.13
	Cr	1.12	0.10
	Mn	6.53	0.38
VOCs (μg/m^3^)	Benzene	1.7	0.54
	Toluene	5.3	1.69
	Ethylbenzene	5.3	1.69
	m,p-Xylene	9.1	2.90
	o-Xylene	5.8	1.85
	Styrene	2.9	0.92

^a^ MDL: Method Detection Limit. ^b^ RSD: Relative Standard Deviation.

**Table 2:** Toxicity information of carcinogens.

Pollutants		Carcinogenic Class	UR ^a^ ((μg/m^3^)^−1^)	Extrapolation Method	Tumor Type	References
Heavy Metals	Ni	A	2.4 × 10^−4^	Additive and multiplicative	Lung cancer	[21]
	As	A	4.3 × 10^−3^	Absolute-risk linear model	Lung cancer	[22,23,24]
	Cd	B1	1.8 × 10^−3^	Only first affected by exposure, extra risk	Lung, trachea, bronchus cancer	[25]
	Cr^6+^	A	1.2 × 10^−2^	Multistage, extra risk	Lung cancer	[26]
VOCs	Benzene	A	2.2 × 10^−6^	Low-dose linearity using maximum likelihood estimates	Leukemia	[27]

^a^ UR: unit risk.

**Table 3:** Toxicity information of non-carcinogens.

Pollutants		Non-Carcinogenic Health Effect	RfC ^a^ (mg/m^3^)	NOAEL ^b^/ LOAEL ^c^ (mg/m^3^)	UF ^d^	MF ^e^	References
Heavy Metals	Mn	Impairment of neurobehavioral function	5.0 × 10^−5^	5.0 × 10^−2^	1000	1	[28,29]
	Cr^6+^	Lactate dehydrogenase in bronchioalveolar lavage fluid	1.0 × 10^−4^	3.4 × 10^−2^	300	1	[30]
VOCs	Benzene	Decreased lymphocyte count	3.0 × 10^−2^	8.2 × 10^0^	300	1	[31]
	Toluene	Neurological effects in occupationally exposed workers	5.0 × 10^0^	4.6 × 10^1^	10	-	[32]
	Ethylbenzene	Developmental toxicity	1.0 × 10^0^	4.3 × 10^2^	300	1	[33]
	m,p-Xylene	Impaired motor coordination (decreased rotarod performance)	1.0 × 10^−1^	3.9 × 10^1^	300	1	[34]
	o-Xylene	Impaired motor coordination (decreased rotarod performance)	1.0 × 10^−1^	3.9 × 10^1^	300	1	[34]
	Styrene	CNC effects	1.0 × 10^0^	3.4 × 10^1^	30	1	[35]

^a^ RfC: reference concentration. ^b^ NOAEL: no observed adverse effect level. ^c^ LOAEL: lowest observed adverse effect level. ^d^ UF: uncertainty factor. ^e^ MF: modifying factor.

**Table 4:** Exposure factors used in this study.

Variables	Mean ± SD ^a^	RME ^b^	References
Body weight (kg)	64.5 ± 12.65	64.5	[38]
Inhalation rate (m^3^/day)	14.62 ± 3.19	18.97	[38]
Exposure duration (day)	300 ± 19	330	[37]
Lifetime (year)	82.7	82.7	[36]

^a^ SD: Standard deviation. ^b^ RME: Reasonable maximum exposure.

**Table 5:** Concentrations of heavy metals and VOCs in PM_2.5_ at each measurement site (heavy metals: ng/m^3^, VOCs: ppb).

Heavy Metals	Measurement Sites	Mean ± SD ^a^	RME ^b^	*p*-Value ^c^	VOCs	Measurement Sites	Mean ± SD ^a^	RME ^b^	*p*-Value ^c^
Ni	A	7.85 ± 17.26	32.37	*p* > 0.05	Benzene	A	3.42 ± 4.29	6.75	*p* > 0.05
	B	9.51 ± 26.89	26.94			B	10.32 ± 27.82	69.31	
	C	8.93 ± 18.33	37.17			C	3.69 ± 4.10	6.83	
	D	23.34 ± 73.44	121.08			D	3.43 ± 2.62	7.99	
	E	18.18 ± 40.74	126.90			E	6.35 ± 19.37	9.95	
As	A	8.08 ± 6.03	20.25	*p* > 0.05	Toluene	A	34.21 ± 31.04	93.72	*p* < 0.05
	B	6.93 ± 5.10	17.12			B	34.01 ± 36.49	82.38	
	C	8.76 ± 6.10	19.71			C	75.78 ± 86.98	224.47	
	D	9.17 ± 7.08	23.45			D	63.86 ± 99.31	300.89	
	E	9.40 ± 7.66	20.92			E	36.85 ± 43.10	108.70	
Cd	A	1.27 ± 0.54	2.12	*p* < 0.05	Ethylbenzene	A	10.57 ± 90.36	28.83	*p* > 0.05
	B	1.21 ± 0.42	2.13			B	20.69 ± 32.58	77.31	
	C	1.10 ± 0.36	1.07			C	29.50 ± 97.81	58.72	
	D	1.09 ± 0.33	1.08			D	14.56 ± 13.55	39.48	
	E	1.70 ± 1.13	4.13			E	16.24 ± 18.40	46.87	
Cr	A	13.10 ± 14.31	3.13	*p* < 0.05	m,p-Xylene	A	21.96 ± 40.27	59.65	*p* > 0.05
	B	10.62 ± 12.43	24.29			B	40.31 ± 65.96	123.93	
	C	20.31 ± 28.33	80.92			C	59.73 ± 244.61	114.35	
	D	38.19 ± 112.52	48.96			D	24.96 ± 23.49	74.52	
	E	15.82 ± 28.35	33.38			E	31.95 ± 31.66	90.06	
Mn	A	19.30 ± 19.28	46.55	*p* > 0.05	o-Xylene	A	8.55 ± 11.17	22.22	*p* > 0.05
	B	27.15 ± 19.37	73.55			B	16.06 ± 22.05	47.93	
	C	29.98 ± 42.00	126.31			C	30.35 ± 155.45	44.09	
	D	31.22 ± 29.01	77.28			D	9.80 ± 8.32	25.35	
	E	25.53 ± 48.83	121.38			E	14.34 ± 12.99	38.55	
-					Styrene	A	0.63 ± 0.81	2.42	*p* > 0.05
						B	0.70 ± 0.67	1.64	
						C	1.40 ± 5.28	2.14	
						D	0.68 ± 0.86	1.94	
						E	0.59 ± 0.86	1.74	

^a^ SD: Standard deviation. ^b^ RME: Reasonable maximum exposure. ^c^ F-Test: ANOVA results for the concentration difference between the measurement sites.

**Table 6:** Carcinogenic risk of heavy metals and VOCs in PM_2.5_ at each measurement site.

Pollutants		Measurement Sites									
		A		B		C		D		E	
		CTE	RME	CTE	RME	CTE	RME	CTE	RME	CTE	RME
Heavy metals	Ni	9.5 × 10^−7^	5.6 × 10^−6^	1.1 × 10^−6^	4.6 × 10^−6^	1.1 × 10^−6^	6.4 × 10^−6^	2.8 × 10^−6^	2.1 × 10^−5^	2.2 × 10^−6^	2.2 × 10^−5^
	As	1.7 × 10^−5^	6.2 × 10^−5^	1.5 × 10^−5^	5.3 × 10^−5^	1.9 × 10^−5^	6.1 × 10^−5^	2.0 × 10^−5^	7.2 × 10^−5^	2.0 × 10^−5^	6.5 × 10^−5^
	Cd	1.2 × 10^−6^	2.7 × 10^−6^	1.1 × 10^−6^	2.7 × 10^−6^	1.0 × 10^−6^	1.4 × 10^−6^	9.8 × 10^−7^	1.4 × 10^−6^	1.5 × 10^−6^	5.3 × 10^−6^
	Cr^6+^	6.3 × 10^−6^	2.1 × 10^−5^	5.1 × 10^−6^	1.7 × 10^−6^	9.8 × 10^−6^	5.6 × 10^−5^	1.8 × 10^−5^	3.4 × 10^−5^	7.6 × 10^−6^	2.3 × 10^−5^
VOCs	Benzene	1.2 × 10^−6^	3.4 × 10^−5^	3.6 × 10^−5^	3.5 × 10^−4^	1.3 × 10^−5^	3.4 × 10^−5^	1.2 × 10^−5^	4.0 × 10^−5^	2.2 × 10^−5^	5.0 × 10^−5^

**Table 7:** Non-carcinogenic risk of heavy metals and VOCs in PM_2.5_ at each measurement site.

Pollutants		Measurement Sites									
		A		B		C		D		E	
		CTE	RME	CTE	RME	CTE	RME	CTE	RME	CTE	RME
Heavy metals	Cr^6+^	6.8 × 10^−3^	2.3 × 10^−2^	5.5 × 10^−3^	1.8 × 10^−2^	1.1 × 10^−2^	6.0 × 10^−2^	2.0 × 10^−2^	3.6 × 10^−2^	8.3 × 10^−2^	2.5 × 10^−2^
	Mn	2.5 × 10^−1^	8.7 × 10^−1^	3.5 × 10^−1^	1.4 × 10^0^	3.9 × 10^−1^	2.4 × 10^0^	4.1 × 10^−1^	1.4 × 10^0^	3.3 × 10^−1^	2.3 × 10^0^
VOCs	Benzene	2.4 × 10^−1^	6.7 × 10^−1^	7.2 × 10^−1^	6.9 × 10^0^	2.6 × 10^−1^	6.8 × 10^−1^	2.4 × 10^−1^	7.9 × 10^−1^	4.4 × 10^−1^	9.8 × 10^−1^
	Toluene	1.7 × 10^−2^	6.6 × 10^−2^	1.7 × 10^−2^	5.8 × 10^−2^	3.7 × 10^−2^	1.6 × 10^−1^	3.1 × 10^−2^	2.1 × 10^−1^	1.8 × 10^−2^	7.6 × 10^−2^
	Ethylbenzene	3.0 × 10^−2^	1.2 × 10^−1^	5.9 × 10^−2^	3.1 × 10^−1^	8.4 × 10^−2^	2.4 × 10^−1^	4.1 × 10^−2^	1.6 × 10^−1^	4.6 × 10^−2^	1.9 × 10^−1^
	m,p-Xylene	6.2 × 10^−1^	2.4 × 10^0^	1.1 × 10^0^	5.0 × 10^0^	1.7 × 10^0^	4.6 × 10^0^	7.1 × 10^−1^	3.0 × 10^0^	9.0 × 10^−1^	3.6 × 10^0^
	o-Xylene	2.4 × 10^−1^	9.0 × 10^−1^	4.5 × 10^−1^	1.9 × 10^0^	8.6 × 10^−1^	1.8 × 10^0^	2.8 × 10^−1^	1.0 × 10^0^	4.1 × 10^−1^	1.6 × 10^0^
	Styrene	1.7 × 10^−3^	9.6 × 10^−3^	2.0 × 10^−3^	6.5 × 10^−3^	3.9 × 10^−3^	8.5 × 10^−3^	1.9 × 10^−3^	7.7 × 10^−3^	1.6 × 10^−3^	6.6 × 10^−3^

**Table 8:** Health risks of target substances predicted through IDW.

Pollutants		ECR				HQ			
		CTE		RME		CTE		RME	
		Max	Min	Max	Min	Max	Min	Max	Min
Heavy metals	Ni	2.8 × 10^−6^	9.5 × 10^−7^	2.2 × 10^−5^	4.6 × 10^−6^	-			
	As	2.0 × 10^−5^	1.5 × 10^−5^	7.2 × 10^−5^	5.3 × 10^−5^	-			
	Cd	1.5 × 10^−6^	9.8 × 10^−7^	5.3 × 10^−6^	1.4 × 10^−6^	-			
	Cr^6+^	1.8 × 10^−5^	5.1 × 10^−6^	5.6 × 10^−5^	1.7 × 10^−5^	2.0 × 10^−2^	5.5 × 10^−3^	6.0 × 10^−2^	1.8 × 10^−2^
	Mn	-				4.1 × 10^−1^	2.5 × 10^−1^	2.4 × 10^3^	8.7 × 10^2^
VOCs	Benzene	3.6 × 10^−5^	1.2 × 10^−5^	3.5 × 10^−4^	3.4 × 10^−5^	7.2 × 10^−1^	2.4 × 10^−1^	6.9 × 10^0^	6.7 × 10^−1^
	Toluene	-				3.7 × 10^−2^	1.7 × 10^−2^	2.1 × 10^−1^	5.8 × 10^−2^
	Ethylbenzene	-				8.4 × 10^−2^	3.0 × 10^−2^	3.1 × 10^−1^	1.2 × 10^−1^
	m,p-Xylene	-				1.7 × 10^0^	6.2 × 10^−1^	5.0 × 10^0^	2.4 × 10^0^
	o-Xylene	-				8.6 × 10^−1^	2.4 × 10^−1^	1.9 × 10^0^	9.0 × 10^−1^
	Styrene	-				3.9 × 10^−3^	1.6 × 10^−3^	9.6 × 10^−3^	6.5 × 10^−3^

## Data Availability

Data are contained within the article.
